# Substitution Attacks: A Catalyst to Reframe the DNA Manufacturing Cyberbiosecurity Landscape in the Age of Benchtop Synthesizers

**DOI:** 10.1089/apb.2023.0035

**Published:** 2024-09-18

**Authors:** Laura Adam, George H. McArthur

**Affiliations:** Built Biotechnologies Inc., Charlottesville, Virginia, USA.

**Keywords:** DNA synthesis, DNA sequencing, biosecurity, bioinformatics, cyberbiosecurity

## Abstract

**Background:**

The advent of easy-to-use benchtop DNA synthesizers has ushered in a transformative era in biotechnology, extending the capabilities of DNA synthesis to nonspecialists. However, this revolution in access to this technology exposes several vulnerabilities, notably in the form of substitution attacks. These attacks exploit the intricate interplay between the digital domain of DNA sequences and the physical reality of synthesis instruments, posing substantial threats to biosecurity.

**Content:**

This article delves deeply into the dynamic and multifaceted landscape of cyberbiosecurity, specifically emphasizing a novel attack vector that evades traditional screening algorithms. To achieve this, the article explores algorithmic approaches designed to screen DNA sequences, shedding light on the vulnerabilities exposed by substitution attacks and recontextualizing the cyberbiosecurity actor landscape in the context of the entire DNA manufacturing process.

**Summary:**

The exploration of cyberbiosecurity brings existing vulnerabilities in DNA screening algorithms to light and sets the stage for future research and policy considerations. By emphasizing opportunities for a comprehensive, multipronged approach rooted in end-to-end practical DNA manufacturing, this study provides a foundation for advancing both knowledge and strategies in the realm of cyberbiosecurity.

**Recommendations:**

This article serves as a clarion call for increased vigilance and innovation in navigating the intricate landscape of cyberbiosecurity. Effectively understanding and mitigating substitution attacks is necessary to safeguard the integrity of synthesized genetic material, particularly in the context of the democratization of DNA synthesis technology.

## Introduction

In 2002, Eckard Wimmer and collaborators achieved a groundbreaking milestone in molecular biology by chemically synthesizing poliovirus cDNA without relying on a natural template.^1^ This achievement not only showcased the remarkable capabilities of synthetic biology but also raised profound questions about the responsible use of such technology. Fast-forward to present day, and the landscape of genetic research has transformed with the widespread availability of synthetic DNA, either through commercial DNA synthesis providers or “printed” by a new generation of user-friendly DNA synthesizers; this empowers researchers to engage in de novo DNA synthesis with unprecedented ease.

Although most DNA synthesis companies work diligently to filter out requests containing any subsequence that could be misused, the increasing availability of benchtop synthesizers raises significant biosecurity risks by putting the technology in the hands of the end users. This study points to the challenges posed by the widespread distribution of DNA synthesis capabilities, highlighting its benefits and dangers. It underscores the urgent need for comprehensive oversight and ethical guidelines to prevent the creation of harmful genetic materials, whether unintentional or deliberate. We draw particular attention to the biosecurity vulnerabilities that benchtop synthesizers present and introduce substitution attacks as a supporting example.

Substitution attacks exploit the inherent trust in DNA synthesis instruments and associated control software, clandestinely manipulating the synthesis process to generate sequences that deviate from the input design. Specifically, a substitution attack involves surreptitiously swapping nucleotides used in a synthesis run in such a way that supplants the order in which the digital DNA sequence had been provided and screened—this results in a different synthetic DNA molecule. The substitution attack could be manifested by hacking the physical cartridge(s) containing the nucleotides or, in some cases, by simply rearranging reagent containers. Conventional screening tools are at risk of falling short in the face of these sophisticated attacks, even in ways that have not yet been publicized, making it imperative to uncover innovative solutions. Leveraging a practical and comprehensive understanding of the DNA manufacturing landscape, our goal is to fortify defense mechanisms against potential biosecurity breaches and ensure the trustworthiness of synthesized genetic material through preventative measures.

In response to the digitization of life sciences research and the increased fusion of biotechnologies with information technologies, cyberbiosecurity emerged as a concept in 2018^2^ that seamlessly incorporates aspects of biosecurity and cybersecurity. The discussion herein sits neatly within the realm of cyberbiosecurity by focusing on security risks at the intersection of the biological and digital domains.^2^ Beginning with a thorough definition of substitution attacks, we explore the landscape of cyberbiosecurity actors in the realm of designer DNA, where the convergence of digital and biological domains necessitates innovative security measures.

## Background: Framing the DNA Manufacturing Cyberbiosecurity Landscape

### Activities Involved in Building Designed DNA

**Figure 1.**
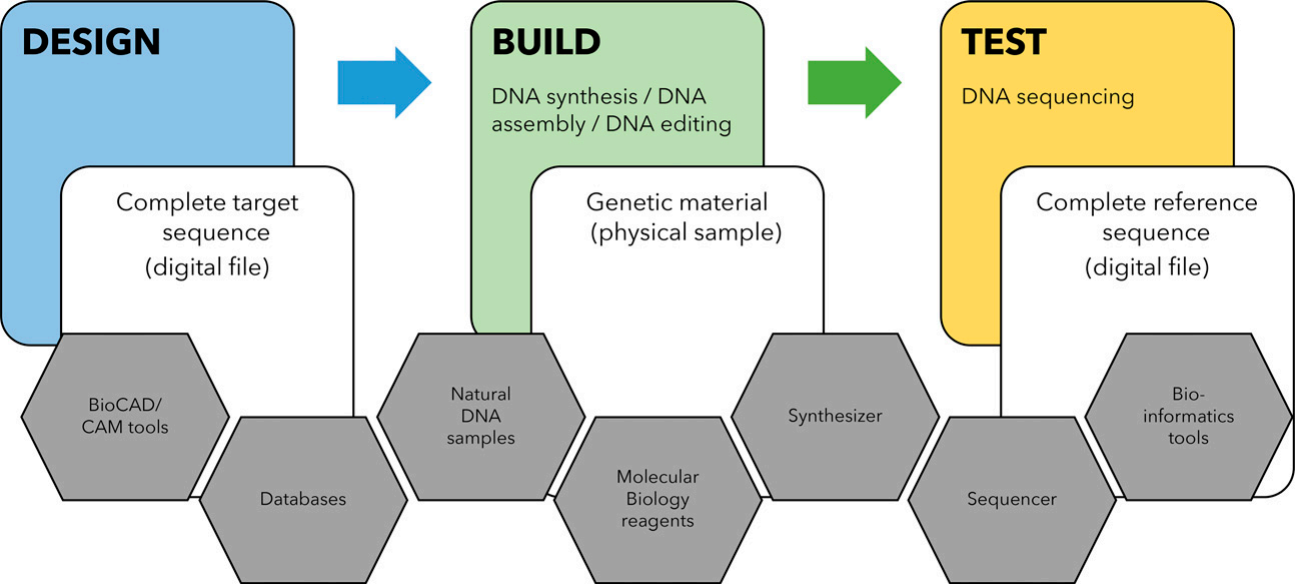
Integrated Workflow: Designing, Building, and Testing Custom DNA molecules.

A comprehensive examination of the manufacturing of designed DNA includes the dissection of the myriad activities that define the entire life cycle of genetic material—from design to verification. The oft-mentioned design–build–test engineering life cycle, in the context of synthetic biology, constitutes a fundamental framework guiding the systematic construction of genetic systems with tailored functionalities as shown in Figure 1.

The process initiates with the *design* phase, often facilitated by specialized software^3–11^ relying on public and proprietary databases. These databases and software tools for genetic design and DNA assembly design play a critical but often overlooked role in generating genetic material with specific functionalities.

In-house benchtop DNA synthesizers^12^ and external, commercial synthetic DNA providers have democratized the synthesis process by providing access to individuals;^13^ this is the first step in the *build* stage of the cycle, allowing researchers to translate digital designs into tangible genetic material at the click of a button. At this point in time, the genetic sequences must be provided explicitly—through a file containing a string of As, Ts, Cs, Gs—although, in the near future, generative design programs ranging from combinatorial design to those realizing the AI promise^14^ will autonomously generate the DNA sequences to build from higher-level specifications without human intervention. Moreover, DNA can be procured through conventional methods involving the extraction of genetic material from natural sources. Common techniques include polymerase chain reaction (PCR) or enzymatic cutting following DNA extraction. PCR-based methods depend heavily on short DNA oligonucleotides (oligos), whose sequences are meticulously designed to precisely target the intended genetic elements, often leveraging information from genomic databases. The manipulation of genetic material goes beyond sourcing DNA (often through synthesis); it extends to the editing phase facilitated by molecular biology reagent kits. Here, researchers employ a variety of molecular tools to precisely modify DNA sequences by introducing novel functionalities, removing undesirable characteristics, or optimizing effectiveness.

After scientists make or edit DNA, they use a process called DNA sequencing to *test* or check if everything turned out as expected. This involves using machines known as DNA sequencers that read the DNA and convert it into data. Availability of user-friendly analysis pipelines and open-source software has democratized the field, enabling researchers across diverse domains to leverage the power of DNA sequencers for a wide range of applications, from basic research to clinical diagnostics. DNA sequencing outputs are typically generated in standard file formats that capture the raw data and processed results. A myriad of bioinformatics tools exist^15–17^ to handle and analyze data generated by DNA sequencers. Typical tasks performed are basecalling to convert raw sequencing signals into nucleotide basecalls, optional demultiplexing, optional assembly of short reads, alignment to a reference sequence, variant calling, and annotations. Basecaller and demultiplexer tools are highly intertwined with the sequencing hardware; therefore, they could be secured by the limited number of DNA sequencing instrument provider. After initial basecalling, which is only concerned with interpreting what the sequencer reads, most bioinformatics tools anticipate a reference sequence. This presents yet another opportunity to screen for sequences, especially for short-read sequencing. Throughout this intricate web of activities, data providers (e.g., NCBI^18^ or ENA^19^) play a fundamental role in supplying the necessary information and genetic sequences for the *design* and *verification* stages.

As the biotech landscape continues to evolve, a nuanced understanding of these interconnected processes—from design software and sequence acquisition to the sourcing of genetic material and reagents and eventually verification through sequencing—is essential for crafting comprehensive policies in the cyberbiosecurity domain. The multifaceted nature of these activities underscores the need for holistic approaches that address the diverse actors and methodologies involved in the production, manipulation, and verification of genetic material in the cyberbiosecurity ecosystem.

### Actors in the Cyberbiosecurity Ecosystem

To comprehend the intricacies of substitution attacks, we must first elucidate the diverse actors operating within the cyberbiosecurity ecosystem. It is a multifaceted domain characterized by an array of actors, each contributing uniquely to the intricate tapestry of applications, molecular biology, informatics, biochemistry, synthetic chemistry, and beyond. Understanding the motivations and roles of the following actors is essential for contextualizing the challenges and opportunities within the realm of cyberbiosecurity.

- Individual researchers and bioengineers: Leverage advanced tools and techniques encompassing genetic design, DNA editing, synthesis, assembly design, amplification, and sequencing to drive scientific discoveries and innovation.
- Regulatory bodies and government agencies: Establish guidelines, policies, and regulations that govern the synthesis, editing, and exchange of DNA. Their role extends to monitoring compliance, assessing potential risks, and adapting regulations to the ever-evolving landscape of synthetic biology.
- Service providers: At the center of attention, DNA synthesis companies must safeguard against potential misuse of synthesized genetic material. Service providers such as DNA sequencing or large-scale DNA prep have similar business since they exchange novel biological samples with their customers.
- Reagent vendors: Provide molecular biology kits for PCR, DNA assembly, and Next-generation synthesizing (NGS) library preparation. These kits equip researchers with the essential tools for amplifying DNA, assembling genetic constructs, executing genome editing, and preparing libraries for next-generation sequencing.
- Instrument vendors: Sequencers and synthesizers enhance accessibility and autonomy by placing the technology in the hands of their customers but also necessitate vigilant oversight to prevent potential security breaches.^20^
- Software and data providers: Supply genetic information for the design phase, including databases offering curated genetic sequences, proprietary databases integrated into design software, and open-access repositories that enrich the diversity of available genetic data. Because Select Agents and Toxins (SAT) sequences are relevant in scientific research, particularly in areas such as biodefense, public health, and the study of infectious diseases, they are also found in public databases.^21^
- Ethical hackers and cybersecurity efforts: Critical in fortifying cyberbiosecurity and identifying vulnerabilities in software, synthesizers, and databases, thereby contributing to the development of resilient cybersecurity measures within the ecosystem.

In addition to this list, the dynamics of insider threats exhibit significant variations depending on whether the technology is operated by *commercial actors* or accessible to *end users*. When managed by commercial actors, the conventional security issue of insider threats persists, encompassing the potential for both unwitting insiders and those with malicious intent. Nefarious actors might infiltrate or manipulate systems from the outside, and the risk of coerced insiders inadvertently revealing vulnerabilities remains. However, the scenario transforms when the technology becomes accessible to the public. In this context, the primary concern centers around bad actors with malicious intentions among end users, as they wield the technology for potential misuse. This distinction emphasizes the necessity for a nuanced examination of insider threats within the cyberbiosecurity ecosystem, recognizing the diverse actors and their respective roles, motivations, and potential risks. It is crucial to acknowledge that, in one case, insider threats constitute a general problem, while in another, it necessitates domain-specific considerations.

As the cyberbiosecurity ecosystem continues to evolve, collaboration and effective communication among these actors become essential for fostering a secure and ethically sound environment for genomic research and synthesis.

### Current Solutions and Regulatory Frameworks

The cyberbiosecurity landscape is fortified by an array of current solutions and regulatory frameworks aimed at safeguarding the biotechnology industry by mitigating potential risks. This segment of the analysis focuses on the Unites States and delves into the oversight frameworks currently employed by DNA synthesis companies and the regulatory frameworks guiding their operations.

- The U.S. Department of Health and Human Services (HHS) provides guidelines for screening synthetic DNA sequences to prevent the synthesis of potentially harmful genetic material. It emphasizes the responsibility of DNA synthesis providers in verifying the legitimacy of customer orders.^22,23^
- The National Institutes of Health (NIH) provides a comprehensive framework for overseeing research involving recombinant DNA and synthetic nucleic acid molecules and enforces safety practices and containment measures to prevent accidental release.^24^
- The Export Administration Regulations (EAR) ensures compliance with international export control regulations to appropriately oversee cross-border transactions pertaining to dual-use biological equipment and genetic material with potential security implications identified through control lists. For the context of our landscape analysis, it is noteworthy to point out that in 2021, EAR added nucleic acid assemblers and synthesizers as well as their operational software.^25^

Academia and government have actively developed since the 1974 Asilomar voluntary moratorium on the rapidly developing practice of recombinant DNA, the role of biosafety and biosecurity experts, and established best practices and guidance for institutions involved in synthetic genomics.^26^ As such, these regulatory frameworks collectively aim to establish guidelines, best practices, and oversight mechanisms to address the potential risks posed by the synthesis and manipulation of genetic material. The emphasis is on preventing accidental releases, ensuring the responsible use of synthetic biology tools, and adapting to the evolving landscape of biotechnology. Researchers, institutions, and DNA synthesis providers are expected to adhere to these frameworks to uphold the safety, security, and ethical standards in the field of synthetic biology.

As a product of history, biosecurity measures have focused on controlling *physical* access to select agents and toxins and, as a result, are reactionary when faced with the dematerialization brought by DNA synthesis. The primary onus for biosecurity has fallen on DNA synthesis providers, serving as custodians of safe technology use. However, the solutions employed can be circumvented when the technology is directly accessible to the end users, as it is with benchtop synthesizers. In our exploration of the practical landscape of DNA manufacturing, as summarized in Table 1, our extensive survey revealed striking gaps in biosecurity measures. Specifically, the verification process involving DNA sequencing actors, bioinformatics tools, and the software and data ecosystem employed in the design phase represents an untapped area for substantial enhancement in biosecurity protocols.

**Table 1.** Landscape of designed DNA manufacturing

Activity	Providers	Oversight
Design	Software	Best Practices
Editing DNA	Molecular Biology Reagents	Export Control
Sourcing DNA	Nature, Laboratory	Export Control
Sourcing DNA	Custom DNA synthesis	Synthesis Screening Framework
Sourcing DNA	Synthesizer	Synthesis Screening Framework
Verifying DNA	Sequencer	Export Control
Verifying DNA	Sequencing reagents	*None*
Verifying DNA	Third-party sequencing services and bioinformatics tools	*None*
Design	Data providers	*None*

## Substitution Attacks: A New Challenge in Cyberbiosecurity

With an established foundation, attention turns to the crux of this analysis—the substitution attack. This section explicates the novel threat posed by substitution attacks, elucidating the mechanisms through which attackers manipulate the DNA synthesis process to generate sequences that are divergent from the intended design. By understanding the nuances of these attacks, we lay the groundwork for evaluating their potential impact on the current cyberbiosecurity paradigm.

### Introducing the Substitution Attacks in DNA Synthesis

**Figure 2.**
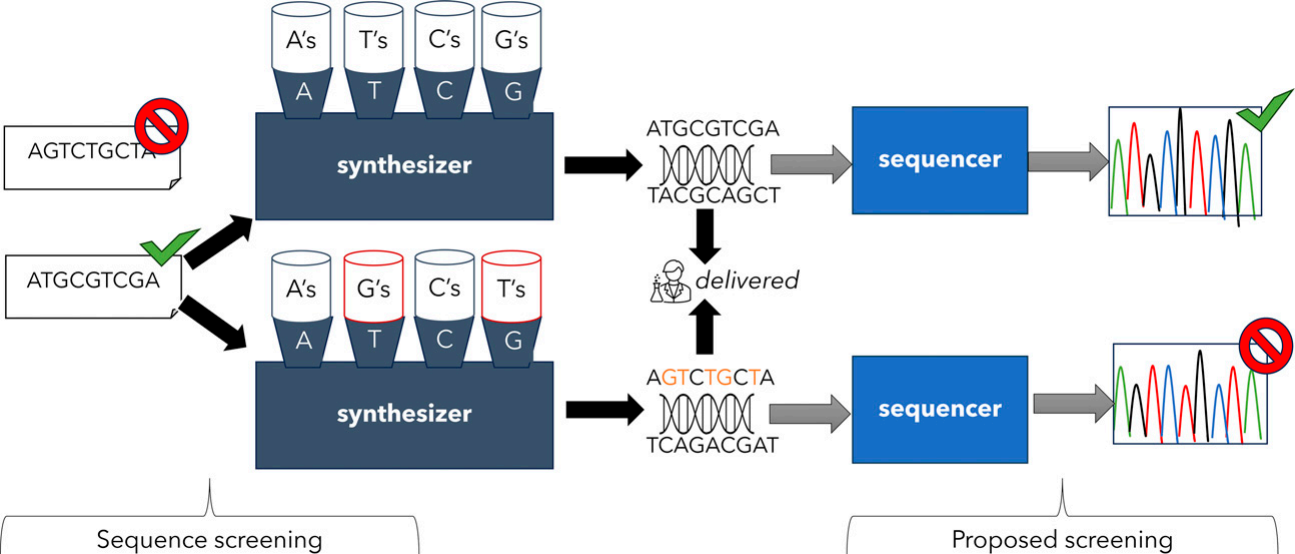
Challenges and opportunities in light of the synthesis substitution attack.

The substitution attack within the context of synthetic biology involves a deliberate manipulation of DNA synthesis processes to evade existing screening algorithms and create genetic material that may be restricted or flagged. In a hypothetical scenario pictured in Figure 2, let us consider a theoretical DNA sequence, AGTCTGCTA, which is identified and flagged as a potentially risky sequence, and therefore, cannot be synthesized using current screening measures. However, nefarious actors exploiting the synthesis substitution attack could strategically alter the DNA synthesis process. For instance, they could physically swap all guanine (G) nucleotides with thymine (T) nucleotides going into the synthesizer and provide an ostensibly benign input sequence (in this case, ATGCGTCGA), circumventing any screening that would have otherwise flagged the problematic sequence. Reagent bottles can be swapped on phosphoramidite DNA synthesis instruments such as Biolytic’s Dr. Oligo^27^ or Cytiva’s OligoPilot.^28^ Instruments based on enzymatic DNA synthesis promise to synthesize dsDNA over 5 KB in the near future.^20^ For instance, DNA Script’s enzymatic oligo benchtop synthesizer (SYNTAX) is loaded through color-coded spaces for the nucleotide containers (4 “inks”),^29^ we hypothesize the “ink” cartridges can be placed in a different order than intended. Either someone could replace what is directly inside the cartridge and keep the cartridge in the expected order containing the wrong nucleotide, or if the cartridges are not differentiated in any specific way, someone can load them in a different order. As a result, the synthesizer proceeds to create the DNA molecule corresponding to the problematic sequence AGTCTGCTA (which would have otherwise been flagged), successfully evading the initial screening measures. This manipulation underscores the vulnerability introduced by the synthesis substitution attack, allowing the creation of genetic material that bypasses existing safeguards and potentially poses security risks.

We have provided a specific example in the supplementary material to illustrate just how effective substitution attacks are at evading BLAST-based screening approaches. Starting with a protein coding sequence (CDS) for blue chromoprotein obtained from the Registry of Standard Biological Parts,^30^ we performed a simple A<>G nucleotide swap. An initial BLASTn search of the National Center for Biotechnology Information (NCBI) database found numerous 100% matches to the original CDS. However, a BLASTn search of the same database, using the same parameter values, was unable to return any hit for the substituted sequence. Of course, the additional swap of T with C would further confound existing screening approaches, making it virtually impossible to identify problematic sequences.

### Evaluating the Impact of Substitution Attacks

A pivotal facet of this analysis involves assessing the impact of substitution attacks on the established cyberbiosecurity ecosystem. This entails a comprehensive examination of potential vulnerabilities in existing screening algorithms, the efficacy of current regulatory frameworks in mitigating such threats, and the overarching consequences for the integrity of synthesized genetic material.

There is an urgent requirement to expand sequence screening protocols to encompass all DNA sequence permutations. Such a broadening of scope would significantly strengthen existing measures to guard against synthesis substitution attacks. However, it is essential to recognize that such an expansion would significantly amplify the workload and resource requirements for screening activities. The number of additional sequence variants that would need to undergo screening can be calculated by considering the permutations of nucleotides. For every sequence, there are 24 possible permutations for which to screen (for each of which additionally screening the reverse complement and all six-frame translations is standard procedure).^31^ This significant increase in screening suggests a substantial resource investment, whether in terms of financial resources to run screening tools in parallel or temporal resources if conducted sequentially.

As indicated in a recent article,^32^ the cost associated with screening is not insignificant, especially for companies involved in large-scale gene synthesis activities. This underscores the financial burden and operational challenges faced by companies engaged in DNA synthesis. Regulatory measures could provide avenues for centralized funding to develop advanced screening tools, offering relief for both large and smaller companies grappling with the expenses associated with maintaining and updating screening infrastructure.

The substitution attack concept brings to light an opportunity for collaboration with DNA sequencing stakeholders. Since DNA sequencing is a common method for checking if DNA has been correctly made or edited, partnering with the companies that make sequencing technology could lead to safer and more secure DNA manufacturing. Sequencing is foundational to genetic analysis and could inherently spot any attempts to swap nucleotides, thereby increasing security throughout the genetic engineering pipeline. If we have an effective and robust tool for checking DNA sequences, it could be used at different points throughout the DNA sequencing data analysis. The criteria for a hit should differ from DNA synthesis screening, significantly reducing computing cost. For example, the screening window would be much longer and decision-making could be set up to obviate manual flagging (e.g., by leveraging well-curated databases). This approach becomes tractable since only a small fraction of all sequencing runs, including applications beyond DNA manufacturing, would involve legitimate handling of samples of concern. Using this framework, if a physical sample’s sequencing data raise a flag, it would be possible for any sequencing data to *not* be returned to the user unless previously approved, hindering illegitimate laboratory work. This collaborative approach aligns with the industry’s best interests, promoting enhanced biosecurity without compromising the efficiency of genetic research and development.

Figure 1 serves as a roadmap for researchers, policymakers, and stakeholders navigating the complex terrain of synthetic biology and cyberbiosecurity in the face of the synthesis substitution hack.

## Discussion

### Challenges

In our review of the DNA manufacturing landscape, the analysis extended beyond the scope of substitution attacks to unveil additional missed opportunities that have implications for biosecurity (Table 2) and reassessed the cyberbiosecurity landscape to foster a multipronged approach we believe to be more robust. Drawing from our participation as technical experts on panels, we have observed that the wide range of expertise required for cyberbiosecurity presents distinct challenges. There is the potential to miss opportunities to implement strong screening protocols for DNA produced using benchtop synthesizers. Moreover, the generalized approach of screening all oligos fails to recognize that one of the concrete uses of these oligos involves manipulating extracted DNA. Indeed, the designed DNA sequences are derived from adjacent genomic regions, making them unlikely to trigger alerts within the current sequence screening framework. The identification of another two substantial pitfalls underscores the need for a more nuanced and comprehensive approach to screening.

**Table 2.** Unaddressed risks and current gaps in cyberbiosecurity

Activity	Unaddressed risks	Identified security gaps
Design	Generative DNA sequence design	Sequence Screening (tool)
Sourcing	Sourcing from wild occurrences	Screening (design against SAT databases)
Synthesizing	Synthesizer substitution hack	Tamperproof, Screening (permutations)
Synthesizing	Bypassing screening	Authenticity and Integrity
Verification	Substitution hack	Screening (sequencing)

SAT, select agents and toxins.

In addition, the current design of reagent containers is not tamper proof, nor are there robust screening sequences on the digital side. This creates ample opportunity for both substitution attacks and potentially costly mistakes when synthesis is done incorrectly or has been tampered with by malicious actors.

The decentralized nature of individual research introduces challenges related to standardization and oversight. Governing bodies and regulatory agencies, able to consider the full picture, must implement effective oversight as researchers operate in silos.

The financial implications are significant, due to the resources required to create the screening tools, run the screening tools during sequencing, and oversee and regulate the screening process. With larger-scale operations, this cost can increase rapidly with the increased sequencing possibilities as well as rapidly changing screening tools and infrastructure. Without additional funding, these measures for security become cost prohibitive.

### Recommendations

Screening at the *design* stage not only presents an opportunity to enhance biosecurity but also mitigates the occurrence of false alarms, a concern that significantly impacts the efficiency of the screening process because of time-consuming follow-ups. The current reliance on screening tools that trigger alerts based solely on the final sequence at the synthesis stage often results in false positives. This consumes, valuable time and resources, particularly for the primary supporters of the screening process—DNA synthesis providers. These false alarms may arise when the design algorithm derives a design from controlled sequences, a common scenario in protein engineering. This process frequently involves leveraging well-studied proteins, often those related to SAT, for critical medical research. It is worth noting that one of the authors personally experienced such challenges, where an algorithm generated designs closely resembling controlled sequences, leading to avoidable subsequent investigations. Concretely, file exchange formats, such as SBOL^33^ and GenBank^34^ can store biosafety-related information, which could be used to streamline the communications of flagged regions. Implementing screening measures at the design stage holds the promise of refining the accuracy of alerts, reducing false positives, and streamlining the screening process for enhanced efficiency and productivity within the DNA synthesis ecosystem.

To bolster the security of the DNA *build* process, two perspectives need to be taken into account. On the physical side, tamper-proof instrument and reagent container designs should become the norm. On the digital side, it is possible and imperative to require screening sequences for synthesizers. Indeed, any instrument requires the conversion of the input DNA sequence into hardware instructions through specialized software. Such software could also perform a biosecurity screen and produce an authenticated instrument instructions file if the sequence is valid. Synthesizers would then require digitally signed inputs. Although sequence screening is feasible, there is an urgent requirement to expand these protocols to encompass all DNA sequence permutations. Such a broadening of scope would significantly strengthen existing measures to guard against synthesis substitution attacks.

DNA sequencing is used to confirm or *test* that the DNA molecule that has been built matches the expected sequence. As described above in the context of substitution attack, we believe it is worth leveraging DNA sequencing actors to further prevent misuse of DNA technologies.

By involving a diverse array of actors, from the design phase to verification, we aim to bridge gaps in understanding and collectively enhance biosecurity measures. There are no clear objectives or directives presented to the qualified actors regarding solutions to safeguard biotechnologies. This broader perspective ensures a more effective and resilient defense against potential threats, both nefarious and naive, aligning with the evolving landscape of synthetic biology and the need for dynamic, adaptive security frameworks.

In response to the intricate challenges posed by substitution attacks, a critical examination of potential changes and innovations within the cyberbiosecurity framework is paramount. Rather than prescribing specific solutions, this article serves to uncover underlying issues and provide directional insights and recommendations to address these concerns. The emphasis is on shedding light on key areas that require attention by guiding future efforts in modifying screening algorithms, refining industry practices, and formulating effective policy recommendations. The intention is not to prescribe ready-made fixes but to stimulate thoughtful consideration and discourse on reframing the cyberbiosecurity landscape. A recent Executive Order on the Safe, Secure, and Trustworthy Development and Use of Artificial Intelligence^35^ encompasses “sequence synthesis procurement screening”. This presents a timely opportunity to accelerate the development of robust technical solutions that could alleviate the threat of substitution attacks.

## Summary

In concluding our exploration of the cyberbiosecurity landscape, it becomes evident that safeguarding the integrity of synthesized genetic material requires a collective and comprehensive effort. The advent of substitution attacks highlights the intricate interplay between the digital and physical aspects of DNA synthesis, underscoring the need for innovative security measures. Moving forward, several key considerations and recommendations emerge.

First and foremost, inviting all stakeholders to the table is imperative. A holistic approach to cyberbiosecurity necessitates the inclusion of diverse actors involved in activities ranging from designing and editing to manipulating and constructing custom DNA. The complex nature of these processes requires collaboration among researchers, DNA synthesis providers, regulatory bodies, and cybersecurity experts. This inclusive approach ensures a more nuanced understanding of the challenges and opportunities within the synthetic biology landscape.

We believe the cyberbiosecurity conversation benefits greatly from the input of practitioners and technical experts who can articulate solutions in advisory panels, particularly those with the ability to effectively communicate with policymakers. Bridging this gap is crucial for translating technical intricacies spanning many domains into actionable policies. Incorporating individuals who possess both technical proficiency and the ability to convey complex concepts in accessible terms enhances the synergy between research and policy. Such interdisciplinary collaboration is pivotal for developing robust regulatory frameworks that adapt to the evolving landscape of synthetic biology.

The conclusion drawn from this examination is a call for collective action in light of novel threats such as substitution attacks. The integration of technical expertise, collaboration across stakeholders, and a nuanced understanding of the cyberbiosecurity landscape are essential components of a resilient defense against emerging threats like substitution attacks. As the synthetic biology ecosystem continues to evolve, so must our strategies for ensuring the responsible and secure manipulation of genetic material. This conclusion serves not as an endpoint but as a catalyst for ongoing dialogue, research, and policy development to foster a biosecure future for the field of synthetic biology.
